# The Network Structure of PTSD Symptoms in Children and Adolescents Exposed to Potentially Traumatic Events: A Systematic Review

**DOI:** 10.3390/children12111516

**Published:** 2025-11-09

**Authors:** Alberto Misitano, Annalisa Tarantino, Febe Geddo, Annalisa Oppo, Barbara Forresi

**Affiliations:** 1 Trauma, Resilience, and Adjustment Investigation (TRAIL) Lab, Department of Psychology, Sigmund Freud University, 20143 Milan, Italy; misitano.phd@milano-sfu.it (A.M.); 62103076@mail.sfu.ac.at (A.T.); geddo.ext@milano-sfu.it (F.G.); a.oppo@milano-sfu.it (A.O.); 2 Contextual Behavioral Science CBS-SFU Lab, Department of Psychology, Sigmund Freud University, 20143 Milan, Italy; 3 Istituto Europeo per lo Studio del Comportamento Umano (IESCUM), 43100 Parma, Italy

**Keywords:** PTSD, network analysis, children, adolescents, systematic review

## Abstract

**Highlights:**

**What are the main findings?**

- Physiological reactivity to trauma-related cues emerged as a recurrently central symptom in PTSD networks among youth.
- Robust associations were observed between symptoms belonging to the same PTSD cluster.
- Preliminary evidence suggests a trend toward increased global connectivity with greater time since exposure.

**What is the implication of the main finding?**

- Heightened physiological reactivity deserves specific attention in future studies.
- Further in-depth investigation of contextual factors shaping PTSD network structures is needed.

**Abstract:**

**Background:** Exposure to potentially traumatic events (PTEs) during childhood and adolescence is relatively common and may result in the development of post-traumatic stress disorder (PTSD). Recent studies have demonstrated the utility of the network approach for examining PTSD symptoms. However, to date, no systematic review has focused exclusively on network-analytic evidence from child and adolescent samples, who require developmental specific evidence to inform clinical practice. Therefore, the present review aimed to summarize network-analytic studies investigating PTSD symptoms among trauma-exposed youth. **Methods**: Guided by the PRISMA guidelines, a systematic search for network-analytic studies exploring the symptom structure of PTSD-only in children and adolescents was conducted using PubMed and EBSCOHost. The methodological quality of included studies was assessed using the NIH Quality Assessment Tool for Observational Cohort and Cross-Sectional Studies. **Results**: Twelve studies (*n* = 12498; *k* = 18) were retrieved, with seven rated as of fair quality. Studies examined DSM-IV (*n* = 10) and DSM-5 (*n* = 2) PTSD symptoms in children and/or adolescents exposed to PTEs (mostly natural disasters). Although central symptoms varied, heightened physiological reactivity consistently emerged among the most central. The strongest associations were observed between symptoms within the same PTSD cluster, and preliminary evidence suggests that network connectivity may increase with time since exposure. **Conclusions**: Heightened physiological reactivity to trauma-related cues appears to be a clinically relevant feature of youth exposed to PTEs, warranting consideration in assessment and intervention. Clinical and methodological implications and future directions for pediatric PTSD research are discussed.

## 1. Introduction

Exposure to potentially traumatic events (PTEs) in childhood is a widespread phenomenon. Epidemiological studies indicate that approximately two-thirds of children and adolescents have experienced at least one PTE worldwide [1], defined as a harmful experience involving exposure to actual or threatened death, serious injury, or sexual violence [2]. Furthermore, research shows that children exposed to PTEs are twice as likely to develop a psychiatric disorder compared to those who have not experienced such events [1]. Notably, one in six children exposed to PTEs meets the criteria for a diagnosis of post-traumatic stress disorder (PTSD), with higher rates for those who have been exposed to interpersonal trauma [3,4,5,6]. The prevalence of PTSD among children and adolescents is particularly relevant, with studies reporting rates ranging from 15% to 43% [3]. PTSD encompasses a broad range of clinical manifestations, within which diverse symptom profiles may be observed. Identifying which profiles characterize PTSD in childhood and adolescence, and whether specific profiles can be distinguished, remains a challenge with important implications for treatment.

Most PTSD studies to date have adopted a categorical approach in assessing the disorder, focusing on the presence or absence of the symptoms outlined in the *Diagnostic and Statistical Manual of Mental Disorders* (DSM). However, this approach often fails to capture the complexity and variability of PTSD symptom configurations, as with many other mental disorder [7]. In recent decades, the limitations of the categorical approach have become increasingly evident [8,9,10], leading many authors to advocate for the greater validity of dimensional approaches to psychopathology [11,12]. These alternative models apply continuous dimensions to psychopathological data, enabling the identification of any manifestation of a disorder. However, dimensional approaches, like their categorical counterparts, share the assumption that symptoms are reflexive indicators of a latent, unobserved entity that both causes their onset and explains their co-occurrence as part of a syndrome [13,14,15]. In other words, a latent variable, whether conceptualized categorically or dimensionally, would explain why certain symptoms occur together. To assert that an underlying disorder is the common cause of its symptoms, their covariance should not result from interactions between them; that is, symptoms should be locally independent. However, in many cases, this assumption is violated in mental disorders [16]. For instance, in PTSD, trauma-related memories may trigger intense psychological distress and physiological reactions, such as increased heart rate and excessive sweating, which in turn can lead to the avoidance of situations or stimuli perceived as threatening. Thus, rather than being locally independent, symptoms of mental disorders are often mutually interconnected [17]. This assumption is the foundation for a novel approach for conceptualizing psychopathology: the network approach [17]. According to this model, mental disorders are viewed as emergent properties arising from a system of interacting nodes (i.e., symptoms). In this view, a disorder is conceived as a self-reinforcing network of interacting symptoms, rather than as the manifestation of an underlying latent cause. Each node is examined along its specific connections (i.e., edges) and its relative influence (i.e., centrality) within the overall structure. Centrality is usually estimated based on (1) the number and proximity of direct connections (i.e., closeness centrality), (2) the extent to which a symptom serves as a bridge between two other symptoms (i.e., betweenness centrality), and (3) the absolute strength of its associations, considering all connections in the network (i.e., strength centrality).

Given the high heterogeneity in PTSD symptom combinations [18], the network approach may be particularly well-suited to explore the differential role of each symptom and potentially mutually reinforcing symptom clusters. Indeed, interest in applying network analysis to PTSD has grown rapidly since the initial studies in 2015 [17,19], resulting in a considerable growth of the empirical literature in this field.

The first systematic review of network analyses on PTSD was carried out in 2020 by Birkeland and colleagues [20]. This review synthesized the results of 20 cross-sectional studies, showing high heterogeneity in terms of the nodes’ centrality (i.e., the position of each node within the network [14]) across different networks. However, the symptom “amnesia” was found to have the lowest centrality in many of the networks, whereas the “intrusive and recurrent thoughts of trauma” and “persistent negative emotional state” emerged as highly central in many of the networks based on the DSM-IV and DSM-5 PTSD criteria, respectively. Furthermore, the strongest and most frequently identified edges were between “exaggerated startle response” and “hypervigilance”, between the two avoidance symptoms (of thoughts and activities), and between “decreased interest in activities” and “narrowing of affect”. Building on this systematic review, Isvoranu and colleagues [21] conducted a meta-analysis of network analytic studies on PTSD. Aggregating results from 33 studies, this meta-analysis again revealed substantial heterogeneity across datasets and did not identify a single symptom as having the most central role across networks. Nevertheless, the symptoms “feeling detached”, “intrusive and recurrent thoughts of trauma”, and “physiological reactivity” to trauma cues consistently appeared as the most central. On the other hand, in line with Birkeland and colleagues [20], “amnesia” emerged as the less central symptom across the analyzed networks. Additionally, the three strongest edges were found between “exaggerated startle response” and “hypervigilance”, between the two avoidance symptoms (of thoughts and activities), and between “feeling detached” and “narrowing of affect”.

However, both reviews predominantly included studies conducted on adults (17 out of 20 in Birkeland et al. [20] and 27 out of 33 in Isvoranu et al. [21], respectively). However, children and adolescents tend to exhibit PTSD symptoms in ways that differ from adults [22,23]. Notably, in younger individuals–unlike adults–the cognitive and emotional systems responsible for interpreting life events are still developing, which may lead to substantial differences in how potentially traumatic experiences are appraised and processed [24]. Developmental differences affect both the type and expression of symptoms: in youth, PTSD tends to manifest with higher levels of behavioral dysregulation, more frequent regressive behaviors, sleep disturbances, irritability, generalized fear, and somatic complains, rather than trauma-specific intrusions [25]. Moreover, children and adolescents are more likely to exhibit externalizing symptoms, while adults typically report more cognitive and internalizing symptoms, such as guilt or persistent negative beliefs [25]. As a result, findings based primarily on adult samples may not be directly applicable to youth, which require developmental-specific evidence to inform clinical practice [22]. However, to date, no systematic review has specifically focused on network analytic evidence from children and adolescent samples.

Therefore, the aim of this systematic review was to qualitatively synthesize existing evidence from network analyses of PTSD symptoms in trauma-exposed children and adolescents. Specifically, the review aimed to examine the network structure and connectivity of PTSD symptoms within this population. Findings will be discussed in light of the previous literature and reviews on the topic, most of which have focused primarily on adult samples [20,21]. This may offer insights for tailoring interventions to developmental needs and symptom profiles.

## 2. Materials and Methods

The reporting of this pre-registered (PROSPERO ID: CRD42024619198) systematic review was guided by the standards of the Preferred Reporting Items for Systematic Reviews and Meta-Analyses guidelines—PRISMA [26].

### 2.1. Information Sources and Search Strategy

To identify potentially relevant studies, a systematic online search was conducted on PubMed and EBSCOhost (APA PsycInfo, APA PsycArticles, PSYINDEX, MEDLINE, ERIC) using the following search string: (PTSD OR “posttraumatic stress disorder” OR “post-traumatic stress disorder” OR “posttraumatic stress symptoms” OR PTSS) AND (children OR adolescents OR teenagers OR youth) AND (“network analysis” OR “network approach” OR “network perspective”). The same search string was applied in both databases. No filters were applied to the search (e.g., publication date, language, article type). No hand-searching of journals or snowballing from reference lists was performed. The final search was conducted on 14 November 2024.

### 2.2. Eligibility Criteria

Original English peer-reviewed studies investigating the network structure of PTSD symptoms only, using data directly collected from children and/or adolescents exposed to at least one PTE, were included in the review. In line with the World Health Organization classification, children were defined as individuals aged below 10 years, adolescents as those aged 10–19 years, and youth as those aged 15–24 years. Studies were eligible for inclusion if the sample was primarily composed of children and/or adolescents, based on the reported mean age and standard deviation. Studies that included participants older than 19 were considered eligible only if the majority of the sample fell within the child and adolescent age range. Studies relying on caregiver reports were excluded, as these may significantly differ from self-reports, potentially failing to accurately reflect the child or adolescent’s symptom presentation [27,28]. Systematic reviews, meta-analyses, and opinion articles were excluded. Given the specificity of the topic, no publication date restrictions were applied, allowing for the inclusion of all published studies up to the time of data extraction.

### 2.3. Data Collection Process

Retrieved articles were manually controlled by two independent reviewers to remove duplicates. Then, title/abstract and full-text screening were independently conducted by reviewers. Data extraction was carried out independently by the same two reviewers, who collected detailed information on the general characteristics of each study as well as on the structural properties of the PTSD symptom networks. Discrepancies were resolved through discussion.

### 2.4. Study Characteristics

For the analysis of the characteristics of the included studies, the following data were extracted: (a) authors and year of publication, (b) research design (type of research, number of network analyses conducted), (c) participants characteristics (number of participants, sample type, type of PTE, time elapsed since trauma, mean age, age range and percentage of females), (d) measures (diagnostic manual used, instruments employed to assess PTSD symptoms), and (e) reproducibility (availability of open data, inclusion on analytical script). To assess the reproducibility of the studies, data were considered open if the full dataset was shared, while the script was considered available if the analytical script was provided.

### 2.5. PTSD Networks

For the analysis of the characteristics of the symptom networks developed in the studies, data were first collected on the following parameters: number of network nodes (i.e., how many symptoms were included in the analysis), type of correlation (i.e., the method of estimating the correlation coefficient), type of regularization (that is, the method used to enhance interpretability of networks), stability analysis (i.e., if analyses were conducted to examine stability, and therefore the interpretability of the network), CS-Betweenness, CS-Closeness, and CS-Strength (i.e., the correlation stability [CS] coefficients of centrality metrics) and significance test (i.e., whether statistical procedures were applied to determine the significance of network parameters).

In addition, based on previous systematic reviews of network analyses [20,29], the following three parameters were chosen as characteristic and informative elements of a network: node centrality, node associations, network connectivity.

Node centrality refers to the importance of a node (i.e., a symptom) in relation to the broader network structure and reflects the degree of connection of individual nodes within the network. Greater centrality of a node indicates a stronger overall association with other nodes. The variables for the CS coefficients to be collected across studies refer to the specific measure used by the individual studies to investigate the stability of node centrality. The correlation stability coefficient is the percentage of the sample that can be removed by maintaining a correlation > 0.70 with the original centrality score. Stability coefficients of 0.25 (cut-off for the interpretable centrality indices) indicate a moderate stability, while coefficients of 0.5 are indicators of strong stability [30]. Therefore, studies or specific networks reporting strength CS-coefficients below 0.25 were interpreted with caution and were not emphasized in the cross-study summary of the most and least central nodes in the networks. Specifically, three centrality indices are commonly used to quantify the importance of individual nodes within a network: closeness (i.e., how close a node is to all other nodes in the network), betweenness (i.e., the frequency with which the node is found in the shortest path between two other nodes), and strength (i.e., how well a node is directly connected to other nodes, irrespective of the sign of the relationship). For psychological networks, however, strength is the most frequently evaluated measure of centrality. Therefore, when studies reported multiple centrality indices, strength centrality was considered the primary outcome for cross-study comparisons of symptom importance. This choice aimed to enhance comparability across studies, given the variability in network estimators and statistical methods. Additionally, following the approach adopted by Birkeland et al. [20] for each network, the three symptoms with the highest centrality and the three with the lowest strength centrality in the networks were identified for analysis.

Node association, on the other hand, explains how often two symptoms co-occur together. The strength of the association between symptoms in a network is graphically represented by the thickness of the line connecting two nodes. This variable was used to detect the most robust associations within the networks.

Finally, network connectivity explains how nodes within the structure are interconnected. In particular, global connectivity refers to the overall degree of interconnectedness among all nodes in the network, and is operationally defined as the sum of the absolute edge weights. Conversely, local connectivity captures the direct connections of each node with its immediate neighbors. To identify the overall degree of symptom interconnection of the networks developed by the included studies, understood as the overall degree of interconnection between the network’s nodes, global connectivity was chosen as the key variable.

### 2.6. Quality Assessment

To our knowledge, at the time of writing, there is no specific measure to assess the risk of bias of studies implementing network analysis [20,21]. Nevertheless, given the observational nature of the included studies, three authors independently assessed the quality of included studies using the National Institutes of Health (NIH) *Quality Assessment Tool for Observational Cohort and Cross-Sectional studies* [31] to gain some information regarding the quality of included studies. In cases of uncertainty, consensus was reached through discussion.

## 3. Results

### 3.1. Selection of Studies

**Figure 1:**
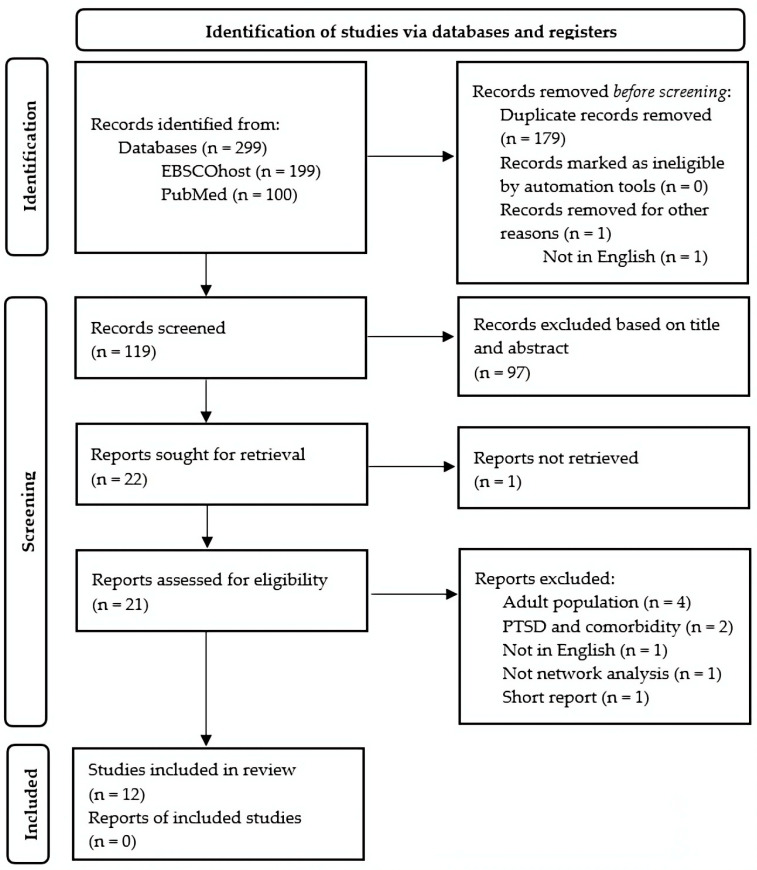
PRISMA Flow Diagram.

The database search yielded 299 potentially relevant studies (100 from PubMed and 199 from EBSCOhost). After the removal of 179 duplicates and the automatic removal on EBSCOhost of one study for language reasons, the remaining 119 studies underwent a two phase selection process. First, articles were screened by title and abstract, resulting in 98 exclusions (including one study that could not be retrieved). In the second phase, the full text of the 21 remaining studies was assessed for eligibility, leading to the exclusion of 9 articles. Consequently, 12 studies were selected for inclusion in this systematic review [32,33,34,35,36,37,38,39,40,41,42,43]. The PRISMA flow diagram of the selection process is shown in Figure 1.

### 3.2. Characteristics of the Studies

The characteristics of the network studies on PTSD symptoms included in the review are summarized in Table 1.

#### 3.2.1. Research Design

Of the 12 included studies, seven used cross-sectional data to estimate PTSD symptom networks [34,35,38,39,41,42], and five conducted longitudinal analysis to assess how the networks changed over time [32,33,36,37,40]. In both cases, most studies estimated more than one network. Specifically, five studies generated two networks, separating participants by sex [34] or age [39,42], comparing two types of exposure to trauma [40] or evaluating symptoms at two time points [33]. Four studies generated three networks, assessing symptoms at three time points [32,36,37] or considering three different types of traumatic events [41]. Overall, this review considered 25 distinct networks of PTSD symptoms.

#### 3.2.2. Participants

Data regarding the participants are summarized in Table 2. The average sample size was 697 participants, with study samples ranging from 211 [41] to 1623 [36]. Seven of the 12 included studies enrolled more than 500 participants [32,35,36,37,40,41,43].

All networks were estimated using community samples of young people including youths recruited from schools in areas affected by natural disasters [32,33,34,35,36,37,41,42], young people aging out the foster care system [43], young refugees [38], young people living in war contexts [39], and young soldiers of the Israel Defense Force (IDF) infantry [40].

In the study on young people exposed to war and armed conflict [39], the overall sample combined participants from six studies conducted in different countries (Burundi, Democratic Republic of Congo, Palestine, Iraq, Tanzania, and Uganda). In the study on young refugees [38], the overall sample included participants from five studies conducted in Germany, with participants mainly originating from Afghanistan, Syria, Eritrea, Somalia, Gambia, Iraq, and Iran.

The potentially traumatic event analyzed in most studies was exposure to natural disasters, particularly earthquakes [32,34,36], but also tornadoes [33], hurricanes [42], and debris flows [37]. Pfeiffer et al. [38] examined a range of migration-related traumatic events but found that natural disasters (along with accidents or serious injuries) were one of the two most reported traumatic events. Yang et al. [41] also included natural disasters in one of their networks, while the other two networks were estimated for disease exposure and accident exposure. Barboza et al. [43] estimated the PTSD network in young people in foster care who had experienced primary (i.e., child abuse and interpersonal violence) and secondary (i.e., home removal) trauma. Scharpf et al. [39] focused on war and armed conflict exposure, while Segal et al. [40] focused on training and armed combat exposure.

Five studies had predominantly female samples, and five predominantly males (e.g., Pfeiffer et al. [38]’s comprised almost exclusively male participants, while Segal et al. [40] had entirely male participants). The study by Cao et al. [34], instead, analyzed two separate samples for females and males, with a higher percentage of females in the overall sample. Only one study [32] did not report the participants’ sex.

Age ranges varied across studies. Some studies (n = 5) focused exclusively on adolescents, and none considered only children. Most included both children and adolescents (n = 7): among these, the majority [35,36,37,38,41] analyzed the total sample, while two [39,42] conducted separate network analysis for children and adolescents. Two additional studies [36,37] did not specify the exact age range but explicitly stated that both children and adolescents were included.

#### 3.2.3. Measurement

As summarized in Table 1, only two studies [38,41] conceptualized the disorder according to DSM-5 criteria, while the majority were based on the DSM-IV criteria for PTSD.

A wide variety of methods was used for collecting symptoms. The most commonly used were the *UCLA PTSD Reaction Index for Children* [44] and the *PTSD Checklist,* PCL [45,46], each administered in three studies. Other tools used were the *Child PTSD Symptom Scale,* CPSS [47], the *Children’s Revised Impact of Event Scale,* CRIES [48], the *Child and Adolescent Trauma Screen,* CATS [49], and the *Composite International Diagnostic Interview Short Form,* CIDI-SF [50].

While in the study of Pfeiffer et al. [38], including several samples of young refugees, all samples were assessed with the CATS for PTSD symptoms, in the study by Scharpf et al. [39], which included multiple samples of youth exposed to war and armed conflict, a variety of assessment tools were used: CRIES-13 for the Palestine sample, the *International Trauma Questionnaire* ITQ [51] for the sample from the Democratic Republic of Congo, UCLA for DSM-IV for the sample from Uganda, UCLA for DSM-5 for the samples from Tanzania and Burundi, and the *Post-traumatic Stress Interview for Children* KID-PIN [52] for the Iranian sample. However, to increase the comparability across studies [7], scores on the CRIES-13, the only one using a 4-point Likert scale (instead of 5-point), were rescaled to the same interval as the other instruments, and only participants with complete data on all 17 DSM-IV symptoms were included in the age-specific samples used to estimate the network.

#### 3.2.4. Reproducibility

Six studies provided open data in the form of reported data matrices [34,35,38,39,40,43], but only two also shared their analytical scripts [34,43].

### 3.3. Quality Assessment

Seven studies (58.3%) were rated as fair quality, and five (41.7%) were classified as low quality [31]. Overall quality was largely influenced by the cross-sectional study design and the assessment of potential confounding variables. The comprehensive assessment is presented in Table 3.

### 3.4. Characteristics of Networks

The results referring to the specific network analyses conducted by the studies are summarized in Table 4. Regarding network estimation methods, most studies used the LASSO graphical algorithm [53] to estimate symptom networks (n = 7 studies), while one study [36] used the FGL, a recent extension of the graphical LASSO. The polychoric correlation method was the most widely used in studies where all variables included were ordinal (n = 7 studies). In terms of network accuracy and stability, the most commonly used stability measure was the case-dropping bootstrap, with the calculation of correlation stability coefficients (CS-coefficients) for the centrality indices. Among studies reporting the CS-coefficients for nodes strength, the mean value was 0.53 (range 0.28–0.75). In addition, four studies reported CS-coefficients for expected influence similar to strength, but taking the sign of the relationship into account [32,34,35,37].

#### 3.4.1. Centrality of Nodes

For all of the included networks, results of node centrality analyses were available. Table 5 illustrates the three most and three least symptoms for each network developed by the studies. Where statistical metrics of symptom centrality were not reported, only the symptoms explicitly described in the text as most or least central were considered. A summary is provided of how frequently each node was identified as the most or least central across studies (Tables S1 and S2).

Aggregated results suggest that among the DSM-IV symptom networks of PTSD [32,33,34,35,36,37,39,40,42,43], three symptoms from the re-experiencing cluster emerged as the most central: the “marked physiological reactivity on exposure to internal or external stimuli related to trauma” (B5) in 13 networks, “flashbacks” (B3) in 11 networks, and “intrusions” (B1) in seven networks. Conversely, “amnesia” (C3) consistently appeared as the least central symptom in 13 networks, followed by “avoidance of trauma-related thoughts” (C1) and “difficulty concentrating” (D3), which were less symptoms in nine and eight networks, respectively.

Among the DSM-5 symptom networks [38,41], the most central symptoms were “difficulty concentrating” (E5), “self-destructive behavior” (E2), and “persistent negative emotional state” (D4), each of which appeared twice among the most central symptoms across the networks. Conversely, “exaggerated startle response” (E4) and “sleeping difficulties” (E6) were the less central symptoms in three and two networks, respectively. Due to the limited number of studies that employed DSM-5-based classification to assess symptoms, it was not possible to identify the third most frequently peripheral symptom within the DSM-5 networks.

#### 3.4.2. Associations Between Nodes

Of the 12 studies examined, six conducted significance tests on differences edge weights, two of which did not provide labels for the reported figures [32,39]. Consequently, the assessment of symptom associations across the reviewed networks was based solely on results reported in the text.

The aggregated results suggest that in the DSM-IV networks, the most robust association was the one between “nightmares” (B2) and “flashbacks” (B3), which appeared among the three most robust edges in nine networks. This was followed, in terms of significance, by the association between “avoidance of trauma-related thoughts” (C1) and “avoidance of activities that evoke memories of trauma” (C2), and the association between “feelings of detachment or estrangement from others” (C5) and “narrowing of affects” (C6), which were identified among the strongest associations in six networks each. In the DSM-5 networks, the strongest association was the one between “avoidance of trauma-related thoughts” (C1) and “avoidance of activities that evoke memories of trauma” (C2) in three networks, followed by the association between “feelings of detachment or estrangement from others” (D6) and “persistent inability to experience positive emotions” (D7) and the association between “concentration problems” (E5) and “sleeping difficulties” (E6), each reported in two networks.

#### 3.4.3. Global Connectivity

Among the included studies, six reported results related to the connectivity of the elaborated networks [34,36,37,39,40]. In the study by Ge et al. [36], global connectivity of the PTSD symptom network was found to increase as a function of time since exposure to the earthquake. Specifically, the global connectivity of the symptom network was significantly stronger at 3 months after the traumatic event (T2 = 5.663) than at 2 weeks (T1 = 5.140), and significantly stronger at 6 months (T3 = 6.094) than at 2 weeks. Similarly, the subsequent study by Liang et al. [37] found that the global connectivity of the PTSD symptom network in the population of young debris flow survivors was not significantly stronger at 15 months (T2 = 7.89) after the traumatic event than at 3 months (T1 = 7.82); however, the global connectivity of the network at 27 months after the event (T3 = 8.42) was significantly higher than the connectivity found at 15 and 3 months, respectively. Comparable results were found in the study by Segal et al. [40], who examined the global network connectivity of PTSD symptoms among adolescents exposed to infantry training and armed combat. Specifically, the authors found significantly stronger global connectivity in the network estimated after 6 months of armed combat exposure (post-combat = 7.92) compared to the network estimated after 6 months of military training (pre-combat = 7.54). Only two studies observed differences in symptom network connectivity by sex and age. Specifically, the study by Cao et al. [34] found a significantly higher global connectivity in the female than the male network (girls’ vs. boys’: 6.62 vs. 5.55), while Scharpf and colleagues [39] reported greater symptom network connectivity in adolescents compared with children (8.04 vs. 6.79), suggesting possible age-related differences in PTSD symptom structure.

## 4. Discussion

While treatment approaches for PTSD have progressed considerably over the past two decades, future gains in effectiveness are likely to depend on acknowledging the heterogeneity of trauma-related symptoms and on designing interventions that can be more precisely adapted to individual patient needs. In fact, the configuration and interconnections of symptoms can vary substantially even among individuals sharing the same PTSD diagnosis. Network analysis represents a key method for advancing our understanding of the differential contributions of PTSD symptoms in trauma-exposed individuals. However, existing systematic reviews and meta-analyses [20,21] have pooled findings from network analyses without distinguishing between adult and youth samples, which may obscure meaningful differences in symptom patterns. Given evidence of developmental-related differences in PTSD onset and expression [22,23,54], this systematic review fills this gap by providing a qualitative synthesis of network analysis conducted on children and adolescents exposed to PTEs. The main results of the present systematic review are threefold: (1) a substantial heterogeneity in node centrality across networks was observed, although consistent patterns of high- and low-centrality symptoms emerged; (2) the strongest associations were generally observed among symptoms within the same PTSD cluster, consistent with the DSM version on which the assessment measure was based; (3) preliminary evidence suggests a trend toward increased global connectivity with greater time since exposure. A more detailed description of these findings and their clinical implications is provided below.

### 4.1. Centrality of Nodes

Despite the heterogeneity of nodes centrality among networks, some symptoms consistently showed high centrality, while others appeared as consistently peripheral across studies. Overall, the findings underscore the prominence of the re-experiencing cluster in the symptoms’ architecture of pediatric PTSD, in line with previous work in the general population [20,21].

Notably, marked physiological reactivity to exposure to internal or external trauma-related cues emerged as the symptom with the highest centrality across DSM-IV networks, aligning with a previous meta-analysis conducted on a predominantly adult sample [21]. The consistent prominence of heightened physiological reactivity across pediatric PTSD networks indicates that this symptom may play a pivotal role in maintaining the overall symptom structure, even in trauma-exposed children and adolescents. Longitudinal studies included in the present review suggest that re-experiencing symptoms tend to remain central or consistently present across networks estimated at different time points after trauma [32,36,37,40], potentially indicating their sustained relevance in the symptom structure of pediatric PTSD over time. These findings are broadly consistent with theoretical models that emphasize conditioned fear responses and impaired extinction learning in individuals with PTSD. Such impairments may hinder the ability to learn that certain cues are no longer associated with danger, thereby sustaining physiological reactivity and contributing to the long-term maintenance of PTSD symptoms [55,56,57]. In youth, developmental neurobiological factors may further amplify these dynamics. The ongoing maturation of autonomic and limbic circuits during childhood and adolescence, combined with still-developing prefrontal regulatory capacities, may increase the salience and persistence of physiologically mediated reactivity and distress in younger individuals. Furthermore, the high centrality of physiological reactivity to trauma reminders may be understood not only as a core clinical symptom of pediatric PTSD, but also as a neurodevelopmentally salient response with broader regulatory implications. Previous research has conceptualized physiological and psychological reactivity to trauma-related cues as potentially supraordinate functions, as reminders can trigger responses not only within the re-experiencing domain, but across multiple PTSD symptom clusters [58]. This can be particularly relevant in children and adolescents, whose neurobiological systems responsible for contextual discrimination, emotional regulation, and extinction learning are still under maturation. Taken together, these findings suggest that heightened physiological reactivity in pediatric PTSD is not only a consistently central symptom across networks, but may also reflect a key neurobiological and cognitive mechanism underpinning the persistence of the disorder in younger populations. However, this pattern should be interpreted cautiously, as most of the included studies employed cross-sectional designs, longitudinal evidence is limited, and centrality metrics alone do not provide evidence of causal or predictive influence.

On the other hand, amnesia consistently emerged as the symptom with the lowest centrality in studies assessing PTSD symptoms according to the DSM-IV criteria, aligning with previous findings from network analyses in the general population [20,21]. Within these prior reviews, among the few studies involving pediatric samples, one study conducted on children [59], not included in the present review, similarly reported a peripheral role of amnesia, further supporting the consistency of this finding across age groups. This is also consistent with prior factor-analytic literature showing weak loadings of this symptom [60]. Taken together, these findings suggest that amnesia is loosely connected to the core symptom network of PTSD and may not represent a central component of the disorder for most trauma-exposed children and adolescents.

In addition, when examining the most and least central symptoms based on DSM-5 criteria, some inconsistencies emerged when compared to DSM-IV networks. Notably, despite consistently low centrality across DSM-IV networks, symptoms such as amnesia and concentration difficulties were found to be among the most central in one [39] and two networks [38,41], respectively, within DSM-5 networks. These observed discrepancies may partly reflect differences in how PTSD symptoms are operationalized and clustered in the two diagnostic systems. For instance, while DSM-IV classifies concentration difficulties within the avoidance/numbing cluster, DSM-5 reassigns this symptom to the hyperarousal cluster, potentially altering its network connectivity profile. Additionally, slight changes in item phrasing or measurement instruments used to capture DSM-5 criteria may influence how symptoms co-occur statistically within the network. Given the limited number of studies employing DSM-5 criteria, these findings should be interpreted with caution. Nonetheless, they underscore the need for future research to investigate whether such differences stem from modifications in diagnostic frameworks, population-specific characteristics, or methodological variations in network estimation procedures.

### 4.2. Stronger Associations

Overall, the results highlight that the strongest associations were predominantly observed between symptoms belonging to the same DSM cluster, in line with previous reviews on PTSD symptom networks [20,21]. The repeated identification of strong intra-cluster associations is theoretically consistent with the modular structure of symptom networks, whereby symptom communities tend to form around shared psychological, emotional, or neurobiological mechanisms [58].

### 4.3. Network Connectivity

The third main result of this review concerns the differences found in connectivity between the analyzed networks. Three interesting findings emerged from the comparison of PTSD symptom networks. First, connectivity in PTSD symptom networks appears to be different when analyzed at different times after the traumatic event: three longitudinal studies revealed greater connectivity between PTSD symptoms in networks estimated at later post trauma time points (months to years after the event) than in networks estimated in the acute aftermath [36,37,40]. This pattern might suggest that young people tend to be more vulnerable to a consolidating of their symptom structure over time. This finding refers to the concept of hysteresis, a phenomenon whereby a psychopathological trauma can cause a wide range of persistent problems even after the triggering stimulus disappears, generating cycles of self-reinforcing symptom activation [17]. The phenomenon of hysteresis can only occur in strongly connected networks [61]. It is therefore plausible that this may be observed in children and adolescents exposed to highly traumatic events where a self-reinforcing symptom network shows a greater global connectivity sometime after the trauma than immediately afterward.

Only two studies observed differences in PTSD symptom networks by sex and age. In one study [34], the global connectivity was stronger in girls’ than in boys’ networks, in line with previous findings on higher PTSD risk for women [3,62], suggesting that girls may exhibit a more strongly connected network of PTSD symptoms, which could increase their vulnerability to hysteresis and the persistence of the disorder. This finding also appears to be in line with the more commonly observed chronic course of PTSD in women and girls [63,64]. While the greater connectivity in girls might point to fewer positive prospects for recovery [65] and suggest the importance of paying particular attention to females during screening and treatment, further studies are needed to clarify the role of sex in PTSD network connectivity.

Another cross-sectional study [39] found that connectivity differed by age, with adolescents showing a greater number of connections between symptoms than children. This comparison suggests that the presentation of PTSD may may vary across developmental stages, with adolescents being potentially more vulnerable to symptom persistence and experiencing stronger cycles of symptom activation. Given that the neural substrates underlying key functions—potentially disrupted in PTSD—undergo significant changes during childhood and adolescence [63,64,65,66], the observed differences in symptom networks might reflect the impact of traumatic stress across developmental stages [67]. Nonetheless, as this evidence comes from a single study, further research is needed to better understand the influence of age on network connectivity.

### 4.4. Limitations and Future Directions

It is important to acknowledge several limitations in this systematic review.

First, precise standards for conducting a review on network analytic studies are not yet available in the literature; for this review, we therefore referred to some recent works systematizing network analysis research applied to psychopathology [20,29]. However, given the recent proliferation of cross-sectional and longitudinal research on symptom networks across several psychiatric disorders, there is an urgent need to develop standards for reviewing studies in this area.

Second, in the absence of established tools for the systematization of results, it was not possible to assess all associations between nodes reported in the included studies. To enable future reviewers to make more precise assessments of the presence or absence of specific associations across studies, each network analysis should ideally provide, as part of its Supplementary Materials, a list of all non-zero associations between nodes identified in the estimated networks. In addition, for the sake of replicability, it is highly desirable that future network studies report bootstrapped confidence intervals for all association weights, centrality stability indices, and correlation stability coefficients. In addition, providing confidence intervals for global connectivity would allow for a more precise estimation of uncertainty at the network level. Finally, researchers should strive to incorporate as many elements of reproducibility as possible, in line with the principles of open science [68].

A further consideration relates to the assessment methods used across studies. In all included networks, PTSD symptoms were assessed via self-report instruments. Although the diagnostic usefulness of self-report instruments has been demonstrated [44], future studies using standardized clinical interviews for the assessment of PTSD symptoms are recommended. Furthermore, as far as research directions are concerned, there is a need to limit the number of clinical measures and diagnostic frameworks used to assess the disorder to a few reliable instruments in order to reduce the wide heterogeneity between studies at the methodological level.

The issue of methodological heterogeneity extends beyond symptom assessment tools and also concerns the procedures used for network estimation. Indeed, considerable variability was observed across studies in terms of the number and selection of nodes included in the network, the correlation metrics used (e.g., polychoric vs. Pearson correlations), and the regularization techniques applied (e.g., graphical LASSO vs. FGL). These methodological inconsistencies may have influenced the comparability of network parameters across studies and should be taken into account when interpreting cross-study findings. To improve cross-study comparability, future research should strive for greater methodological standardization in network estimation procedures, including the use of consistent node sets, correlation metrics, and model tuning parameters.

In addition, we note that differences in instruments and participant age groups may affect measurement equivalence across studies. Symptom items might be interpreted differently depending on developmental stage, potentially impacting network comparability. That said, all measures used in the included studies were validated for the relevant age groups, which partially mitigates this concern. Moreover, since network estimation is sensitive to sample size, it is important to consider that small samples may yield sparse or unstable networks. Nevertheless, the majority of studies included in this review used sufficiently large samples to support robust network modeling.

Another limitation of this review is that almost all studies employed instruments based on the DSM-IV criteria for the diagnosis of PTSD. Although many PTSD criteria remain unchanged across DSM editions, the DSM-5 [2] introduced notable revisions—such as the reorganization into four symptom clusters and the addition of symptoms related to negative cognitions and mood—which, together with further refinements in the DSM-5-TR, limit the generalizability of this review’s findings. To enhance the generalizability of the findings, future network analyses of PTSD in children and adolescents should consistently adopt DSM-5 criteria. Greater clarity in symptom labeling is also needed, as several reviewed studies used ambiguous or overlapping terms that complicate data interpretation. In particular, the label “upset by reminders” was used to refer to the symptom of “intense psychological distress at exposure to internal or external stimuli” in two studies [32,33] and to “marked physiological reactivity to exposure to internal or external stimuli” in another [36].

It is also worth noting that this review focused exclusively on symptom networks, without including potential covariates or comorbid symptoms, in order to enhance accuracy, validity, and specificity of the results. Future research should, however, consider additional factors that may co-occur with PTSD symptoms. For instance, it may be useful to examine whether and how the overall network structure is shaped by external variables, or whether specific PTSD symptoms serve as bridges to potential comorbidities. Moving beyond the assumption of rigid diagnostic boundaries, the network perspective on psychopathology highlights that symptoms of different disorders can interact and mutually reinforce one another across porous boundaries, contributing to comorbidity. For example, recent network-analytic findings suggest that PTSD, anxiety, and depression may not represent entirely discrete disorders [69].

In conclusion, while our review highlights important patterns in centrality and connectivity, we emphasize the need for further investigation of these preliminary findings. Considering that the results of a previous review on centrality rankings and strongest associations among PTSD symptoms [20] were supported by subsequent meta-analytic evidence [21] showing similar results in terms of robust associations and symptom importance, it is desirable that the results of the present review also undergo further evaluation, including tests of network model effect sizes and their corresponding outcomes.

## 5. Conclusions

This review is the first to synthesize the findings from all network analysis studies of PTSD symptoms in children and adolescents conducted to date. Despite substantial variability across studies, physiological reactivity to trauma cues and re-experiencing emerged as the most central symptoms, while amnesia consistently appeared as the least central in trauma-exposed children and adolescents. The strongest associations were predominantly observed between symptoms belonging to the same PTSD cluster. Moreover, PTSD networks in young people may differ in symptom connections depending on time since exposure. These insights may inform more developmentally attuned and targeted approaches to assessment and intervention. Since there is no “one size fits all” approach to PTSD [70], network analyses can help to better conceptualize the disorder in childhood and adolescence and guide the development of interventions tailored to developmental stage, time since trauma exposure, and individual symptom configurations. As an example, directly addressing trauma exposure might not always be required for achieving effective treatment outcomes and recovery in PTSD, whilst arousal and physiological distress may represent the core targets of intervention.

Differences in instruments, diagnostic framework, and network estimation methods constrain cross-study comparability and prevent firm conclusions. Moreover, most available studies are cross-sectional, precluding inferences about temporal or causal relations among symptoms. Additionally, although the network perspective offers valuable clinical implications, centrality measures should be interpreted with caution, as they do not automatically reflect clinical relevance. Recent work has questioned their validity [71,72,73] and highlighted that even peripheral symptoms can be clinically meaningful, since the strengths of statistical association between symptoms is influenced by multiple factors beyond clinical significance. Therefore, centrality and strong symptom associations in observational studies—such as those included in the present review—should be considered as a starting point for future studies to test the differential role of each node in clinical contexts [14].

Despite its limitations, network analysis offers a promising framework for understanding PTSD as a dynamic system of interacting symptoms. By identifying developmentally salient and highly connected symptoms, it may help refine diagnostic formulations and inform interventions tailored to specific age groups and individual symptom profiles.

In conclusion, the present review has synthesized the available network studies of PTSD in children and adolescents, highlighting consistent findings, identifying gaps in the literature, and outlining priorities for future research. By emphasizing both the potential and the limitations of the network approach, it underscores the need for developmentally sensitive conceptualizations of PTSD and for interventions tailored to developmental stage and individual symptom configurations.

## Figures and Tables

**Table 1:** Characteristics of included studies.

Author(s), Year	Type of Study	N of Networks	Version of DSM	PTSD Measure	Open Data	Included Script
An et al., 2021 [32]	Longitudinal	3	DSM-IV	CPSS	No	No
An et al., 2022 [33]	Longitudinal	2	DSM-IV	CPSS	No	No
Barboza et al., 2022 [43]	Cross-sectional	1	DSM-IV	CIDI-SF	Yes	Yes
Cao et al., 2018 [34]	Cross-sectional	2	DSM-IV	UCLA	Yes	Yes
Ferreira et al., 2022 [35]	Cross-sectional	1	DSM-IV	PCL-C	Yes	No
Ge et al., 2019 [36]	Longitudinal	3	DSM-IV	CRIES-13	No	No
Liang et al., 2021 [37]	Longitudinal	3	DSM-IV	UCLA	No	No
Pfeiffer et al., 2019 [38]	Cross-sectional	1	DSM-5	CATS	Yes	No
Russel et al., 2017 [42]	Cross-sectional	2	DSM-IV	UCLA	No	No
Scharpf et al., 2023 [39]	Cross-sectional	2	DSM-IV	NA	Yes	No
Segal et al., 2020 [40]	Longitudinal	2	DSM-IV	PCL	Yes	No
Yang et al., 2024 [41]	Cross-sectional	3	DSM-5	PCL2	No	No

**Table 2:** Participants’ characteristics.

Author(s), Year	Sample(s) Size	Country	Trauma Type	Time Since Trauma	Mean Age	Age Range	% Females
An et al., 2021 [32]	900	China (Wenchuan County)	Natural disaster (earthquake)	1 y 1.5 y 2 y	15	12–19	NR
An et al., 2022 [33]	443 395	China (Yancheng)	Natural disaster (tornado)	3 m 12 m	14	12–17	53 58
Barboza et al., 2022 [43]	636	USA	Mistreatment	NR	16	14–17	58
Cao et al., 2018 [34]	495 373	China (Wenchuan County)	Natural disaster (earthquake)	2.5 y	13	12–16	1000
Ferreira et al., 2022 [35]	662	China	Technological disaster (gas explosion)	NR	13	10–16	44.1
Ge et al., 2019 [36]	1623	China (Baoxing County)	Natural disaster (earthquake)	2 w 3 m 6 m	NR	NR	53
Liang et al., 2021 [37]	1460	China	Natural disaster (debris flow)	NR	12	NR	51.8
Pfeiffer et al., 2019 [38]	419	Germany	Emigration	3 m 15 m 27 m	16	7–21	9.3
Russel et al., 2017 [42]	388	USA (New Orleans)	Natural disaster (hurricane)	3 y	NR	8–13 14–18	51
Scharpf et al., 2023 [39]	412 473	Burundi, DRC, Palestine, Iraq, Tanzania, Uganda	War	NR	11	6–12 13–18	49.8
Segal et al., 2020 [40]	910 725	Israel	Combat training Deployment	6 m 12 m	19	18–24	0
Yang et al., 2024 [41]	622 211 1401	China (Xinjiang)	Accident Natural disaster Disease	NR	14 14 15	10–18	41.48 46.92 53.75

***Note***. NR = not reported. w = weeks. m = months. y = years. All studies were conducted on community samples.

**Table 3:** Quality assessment.

	An et al., 2021 [32]	An et al., 2022 [33]	Barboza et al., 2022 [43]	Cao et al., 2018 [34]	Ferreira et al., 2022 [35]	Ge et al., 2019 [36]	Liang et al., 2021 [37]	Pfeiffer et al., 2019 [38]	Russel et al., 2017 [42]	Scharpf et al., 2023 [39]	Segal et al., 2020 [40]	Yang et al., 2024 [41]
1. Was the research question or objective in this paper clearly stated?	Y	Y	Y	Y	Y	Y	Y	Y	Y	Y	Y	Y
2. Was the study population clearly specified and defined?	Y	Y	Y	Y	Y	Y	Y	Y	Y	Y	Y	Y
3. Was the participation rate of eligible persons at least 50%?	CD	CD	CD	CD	CD	CD	CD	CD	CD	CD	CD	N
4. Were all the subjects selected or recruited from the same or similar populations (including the same time period)? Were inclusion and exclusion criteria for being in the study prespecified and applied uniformly to all participants?	Y	Y	Y	Y	Y	Y	Y	Y	Y	Y	Y	Y
5. Was a sample size justification, power description, or variance and effect estimates provided?	N	N	N	N	N	N	N	N	N	N	N	N
6. For the analyses in this paper, were the exposure(s) of interest measured prior to the outcome(s) being measured?	N	N	N	N	N	N	N	N	N	N	Y	N
7. Was the timeframe sufficient so that one could reasonably expect to see an association between exposure and outcome if it existed?	N	N	N	N	N	N	N	N	N	N	Y	N
8. For exposures that can vary in amount or level, did the study examine different levels of the exposure as related to the outcome (e.g., categories of exposure, or exposure measured as continuous variable)?	N	N	N	N	N	N	N	N	N	N	N	N
9. Were the exposure measures (independent variables) clearly defined, valid, reliable, and implemented consistently across all study participants?	Y	Y	Y	Y	Y	Y	Y	Y	Y	Y	Y	Y
10. Was the exposure(s) assessed more than once over time?	N	N	N	N	N	N	N	N	N	N	N	N
11. Were the outcome measures (dependent variables) clearly defined, valid, reliable, and implemented consistently across all study participants?	Y	Y	Y	Y	Y	Y	Y	Y	Y	Y	Y	Y
12. Were the outcome assessors blinded to the exposure status of participants?	N	N	N	N	N	N	N	N	N	N	N	N
13. Was loss to follow-up after baseline 20% or less?	Y	Y	NA	NA	NA	N	N	NA	NA	NA	Y	NA
14. Were key potential confounding variables measured and adjusted statistically for their impact on the relationship between exposure(s) and outcome(s)?	N	N	N	Y	N	N	N	N	Y	Y	N	Y
**Total Y**	6	6	5	6	5	5	5	5	6	6	8	6
**Overall Quality Grading**	Fair	Fair	Poor	Fair	Poor	Poor	Poor	Poor	Fair	Fair	Fair	Fair

***Note.*** Table summarizing the results of risk of bias assessment using the Quality Assessment Tool for Observational Cohort and Cross-Sectional Studies (National Institute of Health, 2021 [31]). A total number of yes (Y) below 5, between 6 and 9, and equal or above 10 indicates poor, fair, and good quality, respectively. CD = cannot determine; N = no; NA = not applicable.

**Table 4:** Characteristics of PTSD symptom networks.

Author(s), Year	N of Nodes	Correlation	Regularization	Stability Analysis	CS-Betweenness	CS-Closeness	CS-Strength	Significance Testing
An et al., 2021 [32]	17	Polychoric	gLASSO	Yes	0.21 0.13 0.13	0.36 0.36 0.28	0.67 0.67 0.67	No
An et al., 2022 [33]	17	Polychoric	gLASSO	Yes	NR NR	NR NR	NR NR	No
Barboza et al., 2022 [43]	18	Partial	gLASSO	Yes	NR	NR	NR	Yes
Cao et al., 2018 [34]	17	Polychoric	gLASSO	Yes	NR NR	NR NR	NR NR	Yes
Ferreira et al., 2022 [35]	17	Partial	NA	Yes	NR	NR	NR	No
Ge et al., 2019 [36]	13	Partial	FGL	Yes	NR NR NR	NR NR NR	0.75 0.44 0.55	Yes
Liang et al., 2021 [37]	18	Partial	gLASSO	Yes	NR NR NR	NR NR NR	NR NR NR	Yes
Pfeiffer et al., 2019 [38]	20	Polychoric	gLASSO	Yes	NR	NR	0.52	Yes
Russel et al., 2017 [42]	17	Polychoric	gLASSO	No	NR NR	NR NR	NR NR	No
Scharpf et al., 2023 [39]	17	Polychoric	NA	Yes	NR NR	NR NR	0.52 0.44	Yes
Segal et al., 2020 [40]	17	Partial	gLASSO	No	0.13 0.13	0.21 0.21	0.52 0.52	No
Yang et al., 2024 [41]	20	Polychoric	NA	Yes	NR NR NR	NR NR NR	0.36 0.28 0.52	Yes

***Note.*** gLASSO = graphical LASSO; FGL = fused graphical LASSO; CS-coefficients = correlation stability coefficients; NR = not reported.

**Table 5:** Characteristics of PTSD symptom networks central nodes.

Author(s), Year	3 Most Central Nodes	3 Least Central Nodes	3 Most Robust Edges	Network Connectivity
An et al., 2021 [32]	C5, B5, B1 B1, D2, D5 B3, D2, B5	C3, C7, C4 C3, C7, B2 C3, C7, B2	B1-B4, C5-D2, B2-B3 C5-D2, B1-B3, B1-B4 C1-C2, C5-D2, B1-B3	NR
An et al., 2022 [33]	C5, B2, B5 B5, C1, B1	C2, C1, C3 D3, C3, C4	C5-D2, B2-D1, B1-B3 B3-B5, C4-C7, C5-C6	NR
Barboza et al., 2022 [43]	C5, B3, C6	D4, B5, C1	B2-D1, C5-C6, C6-C7	NR
Cao et al., 2018 [34]	C5, B3, B1 B3, B5, C4	D3, C1, C6 D3, C3, D2	C4-C5, B2-B3, B5-C7 B2-B3, C4-C5, C4-C7	6.62 5.55
Ferreira et al., 2022 [35]	B2, C6, C2	C3, C4, B5	C1-C2, C5-C6, B1-B2	NR
Ge et al., 2019 [36]	B1, B4, B3 B4, B3, B5 B3, B5, D2	C3, D3, C1 C3, D3, C1 D3, D5, C1	B3-B4, B1-B4, C1-C3 B3-B4, C2-C3, C1-C3 B3-B4, C2-C3, D2-D4	5.14 5.66 6.09
Liang et al., 2021 [37]	B2, D1, C5 B5, C6_1, B1 B5, B3, D3	C3, C6_2, C1 C6_2, C3, C2 C3, C1, C6_2	C4-C5, B5-C7, B5-D1 C4-C5, B2-B3, C1-C2 C4-C5, B2-B3, B4-D4	7.82 7.89 8.42
Pfeiffer et al., 2019 [38]	B2, E5, B5	E3, D1, C1	B4-B5, E1-E2, B1-B2	NR
Russel et al., 2017 [42]	B5, C2, C6 B2, B5, C6	D4, D2, B2 C6_2, C4, D4	C5-C6_1, B5-C2, B1-B2 C5-C6_1, B4-B5, B5-C2	NR NR
Scharpf et al., 2023 [39]	C1, B5, B3 B1, C1, B5	D4, D2, D3 C3, D3, C4	C5-C6, C1-C2, B2-B3 C1-C2, B1-B4, B2-B3	6.79 8.04
Segal et al., 2020 [40]	B3, B4, D2 B3, B4, D2	C4, D4, B2 D4, C4, C1	C1-C2, B2-B3, C5-C6 B2-B3, D4-D5, C5-C6	7.54 7.92
Yang et al., 2024 [41]	E3, E2, D4 C1, E5, D7 D1, E2, D4	B3, E1, E4 B4, E4, E6 E4, E5, E6	C1-C2, B1-B3, D1-D3 D6-D7, E5-E6, C1-C2 C1-C2, E5-E6, D6-D7	NR NR NR

***Note***. **DSM-IV**: B1: intrusive and recurrent memories of the trauma, B2: distressing and recurrent dreams of the trauma, B3: flashbacks, B4: psychological distress at exposure to internal or external stimuli, B5: physiological reactivity to exposure to internal or external stimuli, C1: avoidance of thoughts of the trauma, C2: avoidance of memories of the trauma, C3: inability to remember an important aspect of the trauma, C4 decreased interest or participation in meaningful activities, C5: feelings of detachment or estrangement from others, C6: narrowing of affect, C7: sense of anticipated future, D1: difficulty falling asleep or staying asleep, D2: irritability or angry outbursts, D3: concentration problems, D4: hypervigilance, D5: exaggerated startle response, C6_1: happiness/love numbing, C6_2: sadness/fear numbing. **DSM-5**: B1: intrusive and recurring memories of trauma, B2: distressing and recurring dreams of trauma, B3: flashbacks, B4: psychological distress at exposure to internal or external stimuli, B5: physiological reactivity to exposure to internal or external stimuli, C1: avoidance of thoughts of trauma, C2: avoidance of memories of trauma, D1: inability to recall an important aspect of the trauma, D2: negative beliefs, D3: distorted blame, D4: persistent negative emotional state, D5: decreased interest or participation in meaningful activities, D6: feelings of detachment or estrangement from others, D7: persistent inability to experience positive emotions, E1: irritable behavior or outbursts of anger, E2: reckless or self-destructive behavior, E3: hypervigilance, E4: exaggerated startle response, E5: concentration problems, E6: sleeping difficulties.

## Data Availability

No new data were created or analyzed in this study. Data sharing is not applicable to this article.

## Supplementary Materials

The following supporting information can be downloaded at: https://www.mdpi.com/article/10.3390/children12111516/s1, Table S1: Summary of Node Centrality Across DSM-IV Networks; Table S2: Summary of Node Centrality Across DSM-5 Networks.
