# Supplementary material for: The Network Structure of PTSD Symptoms in Children and Adolescents Exposed to Potentially Traumatic Events: A Systematic Review
Source: Children (Basel). 2025 Nov 9;12(11):1516. doi: 10.3390/children12111516 (PMC12651395; doi:10.3390/children12111516)
Supplement: Supplementary file 1 [file children-12-01516-s001.zip › children-3934008-supplementary.pdf]

Table S1. Summary of Node Centrality Across DSM-IV Networks.

Symptom	Node	Times among three most central nodes	Times among three least central nodes
Intrusive and recurrent memories of the trauma	B1	7	0
Distressing and recurrent dreams of the trauma	B2	3	3
Flashbacks	B3	11	0
Psychological distress at exposure to internal or external stimuli	B4	4	0
Physiological reactivity to exposure to internal or external stimuli	B5	13	2
Avoidance of thoughts of the trauma	C1	3	9
Avoidance of memories of the trauma	C2	2	2
Inability to remember an important aspect of the trauma	C3	0	13
Decreased interest or participation in meaningful activities	C4	1	6
Feelings of detachment or estrangement from others	C5	5	0
Narrowing of affect	C6	4	1
Happiness/love numbing	C6_1	1	0
Sadness/fear numbing	C6_2	0	4
Sense of anticipated future	C7	0	3
Difficulty falling asleep or staying asleep	D1	1	0
Irritability or angry outbursts	D2	6	3
Concentration problems	D3	0	8
Hypervigilance	D4	0	6
Exaggerated startle response	D5	1	1

Table S2. Summary of Node Centrality Across DSM-5 Networks.

Symptom	Node	Times among three most central nodes	Times among three least central nodes
Intrusive and recurrent memories of the trauma	B1	0	0
Distressing and recurrent dreams of the trauma	B2	1	0
Flashbacks	B3	0	0
Psychological distress at exposure to internal or external stimuli	B4	0	1
Physiological reactivity to exposure to internal or external stimuli	B5	1	0
Avoidance of thoughts of the trauma	C1	1	1
Avoidance of memories of the trauma	C2	0	0
Inability to remember an important aspect of the trauma	D1	1	1
Negative beliefs	D2	0	0
Distorted blame of self or others	D3	0	0
Persistent negative emotional state	D4	2	0
Decreased interest or participation in meaningful activities	D5	0	0
Feelings of detachment or estrangement from others	D6	0	0
Persistent inability to experience positive emotions	D7	1	0
Irritable behavior or outbursts of anger	E1	0	1
Reckless or self-destructive behavior	E2	2	0
Hypervigilance	E3	1	1
Exaggerated startle response	E4	0	2
Concentration problems	E5	2	1
Sleeping difficulties	E6	0	2
